# Cultivation of Human Corneal Endothelial Cells Isolated from Paired Donor Corneas

**DOI:** 10.1371/journal.pone.0028310

**Published:** 2011-12-16

**Authors:** Gary S. L. Peh, Kah-Peng Toh, Fei-Yi Wu, Donald T. Tan, Jodhbir S. Mehta

**Affiliations:** 1 Singapore Eye Research Institute, Singapore, Singapore; 2 Singapore National Eye Centre, Singapore, Singapore; 3 Duke Medical School of Medicine, National University of Singapore, Singapore, Singapore; 4 Yong Loo Lin School of Medicine, National University of Singapore, Singapore, Singapore; University of Missouri-Columbia, United States of America

## Abstract

Consistent expansion of human corneal endothelial cells (hCECs) is critical in the development of tissue engineered endothelial constructs. However, a wide range of complex culture media, developed from different basal media have been reported in the propagation of hCECs, some with more success than others. These results are further confounded by donor-to-donor variability. The aim of this study is to evaluate four culture media in the isolation and propagation of hCECs isolated from a series of paired donor corneas in order to negate donor variability.

Isolated primary hCECs were cultured in four previously published medium coded in this study as: M1-DMEM; M2-OptiMEM-I; M3-DMEM/F12, & M4-Ham's F12/M199. Primary hCECs established in these conditions were expanded for two passages and analyzed for (1) their propensity to adhere and proliferate; (2) their expression of characteristic corneal endothelium markers: Na+K+/ATPase and ZO-1; and (3) their cellular morphology throughout the study. We found that hCECs isolated in all four media showed rapid attachment when cultured on FNC-coated dishes. However, hCECs established in the four media exhibited different proliferation profiles with striking morphological differences. Corneal endothelial cells cultured in M1 and M3 could not be propagated beyond the first and second passage respectively. The hCECs cultured in M2 and M4 were significantly more proliferative and expressed markers characteristics of human corneal endothelium: Na+K+/ATPase and ZO-1. However, the unique morphological characteristics of cultivated hCECs were not maintained in either M2 or M4 beyond the third passage.

The proliferative capacity and morphology of hCECs are vastly affected by the four culture media. For the development of tissue engineered graft materials using cultured hCECs derived from the isolation methodology described in this study, we propose the use of proliferative media M2 or M4 up to the third passage, or before the cultured hCECs lose their unique cellular morphology.

## Introduction

The human corneal endothelium (CE) plays a critical role in the regulation of corneal hydration, maintaining corneal thickness, and keeping the cornea transparent [1], [2]. The human CE has a very limited propensity to proliferate *in vivo* [3], [4]. Hence, in order to replace dead or damaged corneal endothelial cells (CECs), the existing cells spread out to maintain functional integrity and sustain corneal deturgescence [5], [6]. In a situation whereby an individual experiences accelerated or acute corneal endothelial cell-loss due to either accidental or surgical trauma, endothelial dysfunction of the CE layer may occur. This results in their inability to pump fluid out of the stroma, causing stromal and epithelial edema, loss of corneal clarity and visual acuity, and will eventually lead to the clinical condition of bullous keratopathy [2]. The current solution to restore vision is to replace the dysfunctional endothelium with healthy donor CE through a corneal transplant [7].

There is a global shortage of transplant-grade donor corneal tissues, which greatly restricts the number of corneal transplantations performed yearly. This shortage will most likely be aggravated as the demand for corneal transplantation increases along with an aging global population that is enjoying a longer life span [2]. Therefore, considerable clinical interest has been generated for the development of suitable endothelial graft alternatives through cell-tissue engineering, which can potentially alleviate the shortage of corneal transplant material [8]. With the rapid advancement in the field of endothelial keratoplasty, a less invasive key-hole surgery option for the selective replacement of the corneal endothelial layer is now possible, and these procedures include Descemet's Stripping Endothelial Keratoplasty (DSEK) and Descemet's Membrane Endothelial Keratoplasty (DMEK), the latter being a procedure whereby only the DM with attached endothelium is replaced [9], [10], [11]. Such an approach enables the delivery of a thin lamellar corneal graft, making the overall concept of a cell-tissue engineered replacement of the endothelial layer even more appealing. However, in order to facilitate the development of such an endeavor, a robust systematic procedure that enables the propagation and expansion of cultured human corneal endothelial cells (hCECs) *in vitro* becomes very critical. Current published isolation and cultivation methods for the establishment and propagation of hCECs vary greatly between laboratories, some with more success than others [2], [12], [13], [14], [15]. This is partly due to the use of complex serum-supplemented culture media that were developed from different basal medium formulation, and can be partly attributed to donor variations which can be highly variable between donor samples [4], [13]. To our knowledge, no comparison has been made between published culture systems to compare their efficiency in supporting the isolation and long-term cultivation of adherent hCECs isolated from a single donor to discriminate against donor sample variability.

In this study, we utilized a modified two-step “peel-and-digest” procedure for the isolation of hCECs from paired cadaveric research-grade donor corneas. To our knowledge, this isolation strategy is a first report that enabled a valid side-by-side assessment of various serum-supplemented media used in the propagation of isolated hCECs, whilst minimizing potential donor sample variability. Primary hCECs isolated were divided equally and cultured in four previously published culture media [12], [13], [14], [15]. The capacity to establish, as well as to cultivate the primary hCECs in each of the four medium, within the experimental perimeters described in the current study, was assessed. More importantly, this study is done with a goal towards defining and establishing more robust culture methodologies to initiate further development of suitable tissue engineered endothelial graft alternatives.

## Results

### Isolation and establishment of primary culture of human corneal endothelial cells

**Figure 1:**
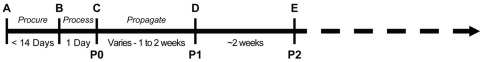
Schematic diagram depicting processes involved in the isolation and propagation of hCECs. **A**: All research-grade corneas used in this study were procured from Lions Eye Institute for Transplant and Research Inc. (Tampa, FL). Research corneas were preserved and transported in Optisol-GS, and were used within 10 days from preservation. **B**: Once received, corneas were washed thrice in an antibiotic, antimycotic wash solution. The DM-CE was peeled and the hCECs were isolated and plated into passage 0 cultures within a day. **C**: Isolated hCECs were seeded and propagated in the 4 culture conditions for up to 4 weeks. **D** and **E**: Confluent cells at each time point were trypsinized using TrypLE Express and seeded at a matched density of 5,000 cells/cm^2^.

**Figure 2:**
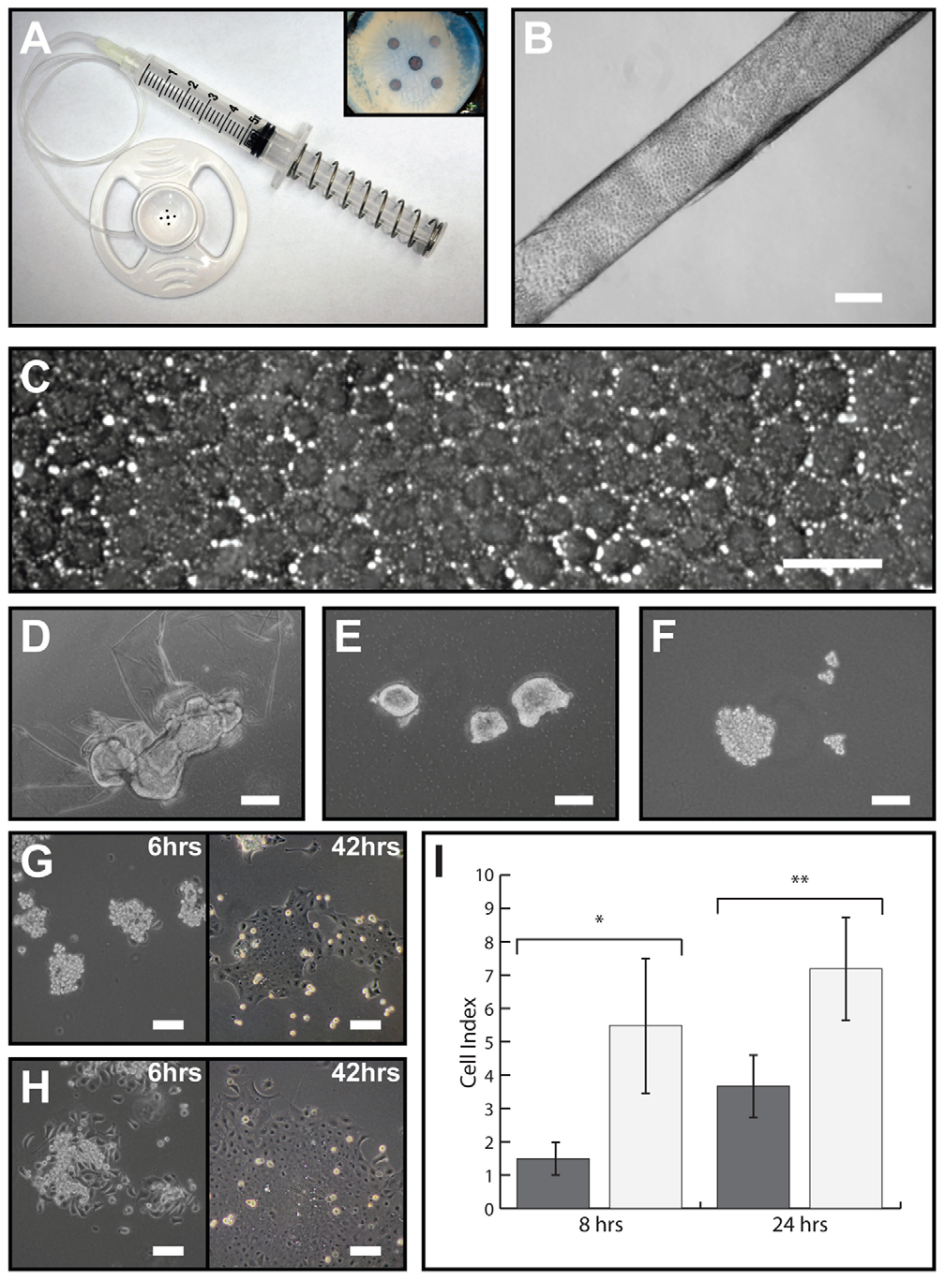
Isolation and establishment of hCECs. **A**: Cornea suction punch. Insert shows a research-grade cornea mounted endothelial side up, stabilized and held relatively firmly in place by the vacuum suction created. **B**: Peel DM-CE layer that spontaneously rolled endothelial side out (scale bar = 200 µm). **C**: High magnification micrograph of the DM-CE layer showing the unique hexagonal morphology of the corneal endothelial cells (scale bar = 50 µm). **D**: Enzymatically dissociation of the DM-CE layer using collagenase (2 mg/mL) for approximately 2 hours resulted in the CE layer slowly displaced off the DM. **E**: Extended dissociation (up to 4 hours) of the DM-CE layer in collagenase fully dislodged the CE from the DM. Interestingly, the CE layer balled-up to form tightly packed CE clusters; **F**: Further dissociation of the CE clusters using TryPLE Express for 5 minutes enable the CE clusters to be loosen into smaller CE clusters for the culture and comparison of hCECs in 4 different culture conditions. **G**: The morphology of hCECs seeded on culture-ware without FNC coating at 6 hrs and 42 hrs as compared to **H**: the morphology of hCECs plated on culture-ware coated with FNC coating mixture at 6 hrs and 42 hrs showed distinctive differences. **I**: The adherence of cultured hCECs (P1 cells; *n* = 3) was also analyzed using xCELLigence real-time impedance-based cell analyzer system and a significant cell index value were observed in hCECs cultured on FNC coated surface at both 8 hrs (*t* = −3.82 ^*^ *p*<0.01) and 24 hrs (*t* = −3.90 ^**^ *p*<0.01). Unless otherwise stated, all scale bars = 100 µm.

The schematic (Figure 1) of the workflow in this study portrays the timeframe taken to procure and process the donor corneas for the isolation and propagation of hCECs. The DM-endothelial layer was carefully harvested with the aid of a vacuum suction holder (Figure 2A). Pure DM-endothelial layer harvested using this methodology, similar to that performed in DMEK donor surgery, rolled spontaneously endothelial side out (Figure 2B). Under phase-contrast microscopy, homogeneous hexagonally shaped CECs could be seen on the harvested DM-endothelial layer (Figure 2C). The use of collagenase for the isolation of hCECs from the DM showed partial isolation after two hours treatment (Figure 2D). Full dislodgement of the CECs from the DM was achieved following extended collagenase treatment of up to 6 hours (varies greatly between donors), which conglomerated into tightly packed CE clusters (Figure 2E). A brief treatment of the isolated CE clusters using TE aided the dissociation of larger CE clusters into smaller clumps, as well as single cells (Figure 2F), which enabled an even distribution of the isolated CECs into the 4 culture conditions. The use of extracellular matrices (ECM) greatly improved the attachment (6 hrs) and expansion (42 hrs) of isolated hCECs cultured on FNC-coated cell culture-wares (Figure 2H) as compared to hCECs grown on uncoated cell culture-wares (Figure 2G). The above observation was also reflected significantly in the adherence of sub-cultured P1 hCECs (*n* = 3) at 8 hrs (*t* = −3.82 ^*^ *p*<0.01; Figure 2I) and 24 hrs (*t* = −3.90 ^**^ *p*<0.01; Figure 2I).

### Morphology of hCECs in the four culture conditions at P0

**Figure 3:**
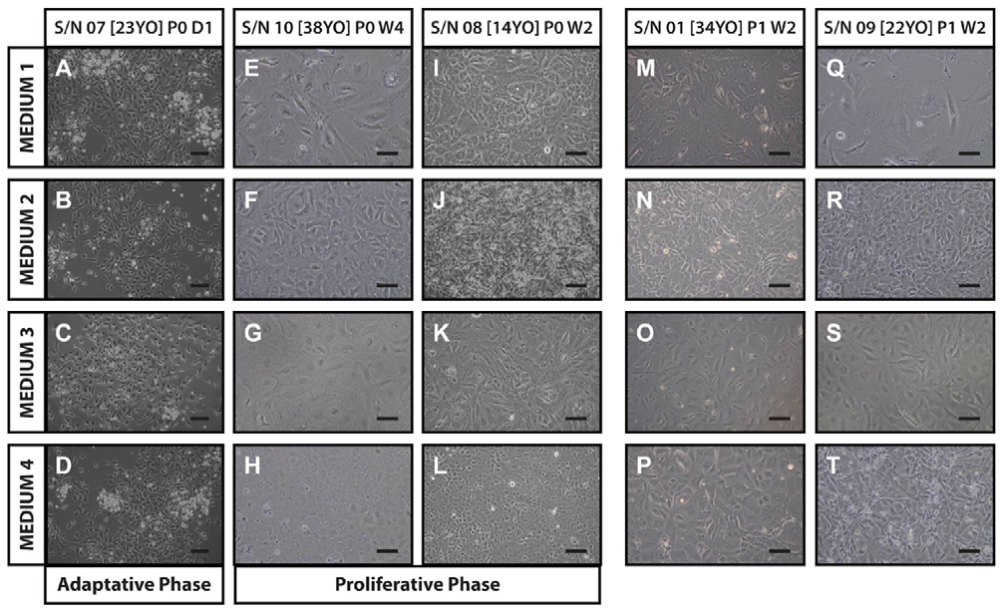
Morphology of cultured hCECs P0 to P1. Representative sets of photomicrographs showing morphology of hCECs at passage 0 and passage 1 cultured in the 4 culture conditions over various time points. **A** to **D**: S/N07 passage 0 day 1 after attachment (adaptive phase). **E** to **H**: S/N10 passage 0 week 4 at the end of the proliferative phase before passaging. **I** to **L**: S/N09, **M** to **P**: S/N01, and **Q** to **T**: S/N09 are passage 1 week 2 cultures derived from three pairs of donor corneas.

The morphology of hCECs established in the four culture conditions (Table 1) was analyzed using phase-contrast microscopy (Figure 3). Isolated hCECs showed no striking morphological differences within this adaptive phase as observed 24 hours after plating (Figure 3A to 3D). However, the cultured hCECs displayed variations in cell morphology during and throughout the proliferative phase at P0 (Figure 3E to 3L). Specifically, hCECs cultured in M1 were the least proliferative, and cells were the largest and most irregular in shape (Figure 3E; *n* = 5 3565.92±1855.33 µm^2^; COV: 52.03; Table 2). M2, a well-characterized medium for the culture of hCECs, supported the proliferation of hCECs. Isolated hCECs grown in M2 were generally small, compact in sizes, and were mostly polygonal (Figure 3F; *n* = 5; 1073.61±161.18 µm^2^; COV: 15.01; Table 2). Isolated hCECs cultured in M3 appeared to be more proliferative than those cultured in M1, but were far less proliferative when compared to hCECs cultured in M2 or M4. Cell sizes of hCECs cultured in M3 were relatively larger, but maintained a homogenous appearance (Figure 3G; *n* = 5; 2304.76±669.21 µm^2^; COV: 29.04; Table 2). M4, another well-characterized culture medium for the growth of hCECs, supported the expansion of the isolated hCECs in the proliferative phase. The cultured hCECs grew well to form a high cell density monolayer, and their polygonal morphology was retained (Figure 3H; *n* = 5; 952.22±189.15 µm^2^; COV: 19.86; Table 2). Statistical analysis using two-way ANOVA with post-hoc Bonferroni test for multiple comparisons showed significance cell-size differences in hCECs cultured in: M1 & M2, ^*^ *p*<0.01; M1 & M3, ^**^ *p*<0.01; M1 & M4, ^†^ *p*<0.01; M2 & M3 ^‡^ *p*<0.01; as well as M3 & M4, ^§^ *p*<0.01 (Table 2). It should be noted that in some of our observations, hCEC-cultures that were established from younger donors (Figure 3I to 3L) appeared to be more proliferative across all the four culture conditions compared to those cultures established from older donors. For example, the hCECs derived from a 14 year-old donor, cultured in M1 (Figure 3I) and M3 (Figure 3K) formed a relatively compact monolayer and appeared more homogeneous and regular in the overall cell sizes and cell shapes. Interestingly, these same hCECs that were cultured in M2 medium proliferated extremely rapidly in a disordered manner and appeared to have lost their unique polygonal morphology as well as contact inhibition (Figure 3J). In comparison, the M4 expanded CECs that were isolated from the same donor formed a homogenous monolayer of cells with hexagonal morphology (Figure 3L).

**Table 1:** Supplemented media used in the culture of human corneal endothelial cells.

Basal Medium	Serum	Growth Factors & Supplements	Reference
[M1]DMEM	10%	2 ng/ml bFGF50 U/ml penicillin50 µg/ml streptomycin	Ishino et al., 2004 [12]
[M2]Opti-MEM-I	8%	20 ng/ml NGF5 ng/ml EGF20 µg/ml ascorbic acid200 mg/L calcium chloride100 µg/ml pituitary extract50 µg/ml gentamicin1× antibiotic/antimycotic0.08% chondroitin sulphate	Zhu and Joyce, 2004 [13]
[M3]SHEMHam's F12 & DMEM (1∶1 ratio)	5%	0.5% DMSO2 ng/ml EGF5 µg/ml insulin5 µg/ml transferrin5 ng/ml selenium0.5 µg/ml hydrocortisone1 nM cholera toxin50 µg/ml gentamicin1.25 µg/ml amphotericin B	Li et al., 2007 [14]
[M4]F99Ham's F12 & M199 (1∶1 ratio)	5%	20 µg/ml ascorbic acid20 µg/ml bovine insulin2.5 µg/ml transferrin0.6 ng/ml sodium selenite10 ng/ml bFGF	Engelmann et al., 1989 [15]Engelmann and Friedl, 1995 [24]

**Table 2:** Cultured P0 hCECs cell sizes and coefficient of variation at confluence.

Culture Medium	Cell Size±SD (µm^2^)	Coefficient of Variation
[**M1**]	3565.92±1855.33* ^/^ ** ^/^ †	52.03
[**M2**]	1073.61±161.18* ^/^ ‡	15.01
[**M3**]	2304.76±669.21** ^/^ ‡ ^/^ §	29.04
[**M4**]	952.22±189.15† ^/^ §	19.86

Comparison of cell sizes cultured in the four media (*n* = 5) was performed using two-way ANOVA followed by post-hoc Bonferroni test for multiple comparisons, and significance was achieved between M1 & M2,

*p<0.01; M1 & M3,

**p<0.01; M1 & M4,

† *p*<0.01; M2 & M3,

‡ *p*<0.01; M3 & M4,

§ *p*<0.01.

### Morphology of hCECs in the four culture conditions at P1 and P2

The morphological differences of the hCECs (*n* = 8) passaged in their four respective culture media became more evident by the second week of the first passage as shown in two representative hCEC-cultures derived from aged 34 year-old donor (Figure 3M to 3P), and aged 22 year-old donor (Figure 3Q to 3T). Passaged CECs cultured in M1 were generally sparse even though the same numbers of initiating cells were seeded. These cells remained to be the largest and most irregular in shape amongst the four conditions (Figure 3M and 3Q). Most of the hCECs cultured in M2 maintained their morphological profile of cells that are generally small with a small variance in sizes (Figure 3N and 3R). The hCEC cultures that were propagated in M3 were larger in cell sizes, but remained consistent in the overall cell shape and formed confluent cultures with lower cell density (Figure 3O and 3S). The general expansion profiles and structural morphology of hCECs cultured in M4 (Figure 3P and 3T) were comparable to hCECs cultured in M2.

### Proliferation and expansion prolife of cultured hCECs

**Figure 4:**
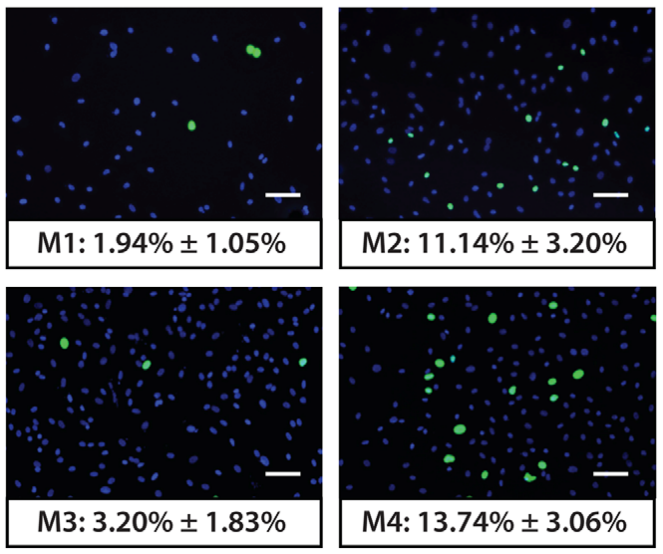
Proliferative capacity of hCECs in the four culture media. Percentages of proliferative P1 hCECs were visualized using the Click-iT EdU assay (*n* = 8). Statistical analysis using chi-squared comparisons with Yates correction showed a significantly greater proportion of proliferative cells in M2 and M4 cultured hCECs compared to M1 and M3 cultured cells (*p*<0.01).

The percentage of proliferative hCECs in each of the four culture media was assessed using a Click-iT Alexa Fluor 488 EdU incorporation assay (*n* = 8). Passaged hCECs subjected to the four culture conditions showed different proliferation rates as judged by the percentage of EdU incorporated cells (Figure 4). Cultured hCECs were less proliferative in M1 (1.94%±1.05%) and in M3 (3.20%±1.85%) compared to M2 (11.14%±3.20%) and M4 (13.74%±3.06%), which were able to support the continual expansion of hCECs. Statistical analysis using chi-squared comparisons with Yates correction showed a significantly greater proportion of proliferative cells in M2 and M4 cultured hCECs compared to M1 and M3 cultured cells (*p*<0.01). This observation was consistent for all of the cornea pairs isolated from donors younger than 38 year old (results not shown). Although the proliferative rates of hCEC-cultures established in M1 and M3 were slower than those in M2 and M4, each sample set was passaged at the same time point when both M2 and M4 reached confluence.

At the point of passage, cultured hCECs in each condition were dissociated into single cells, and cell counts were performed. Equal numbers of hCECs were then plated for each of the four conditions and cultured for at least 2 weeks in their respective culture medium. For this study, passaging was performed, where possible, until the third passage. The absolute numbers of hCECs obtained from each culture condition during each passage derived from manual cell counts were tabulated for comparison using a non-parametric Kruskal-Wallis test with pair wise comparisons corrected for multiple comparison using Mann-Whitney U test (Table 3). At the end of P0, results of cell counts obtained from 6 sample sets showed that hCECs cultured in M2 expanded the most (123,625±11,813 cells), followed by cells that were cultured in M4 (102,832±35,534 cells), and these were statistically significant when compared to M1 (20,458±10,862 cells; ^*/†^ *p*<0.01) and M3 (26,208±16,335 cells; ^‡/§^ *p*<0.01) respectively (Table 3). At the end of P1, the results obtained were similar to those of P0 where hCECs cultured in M2 (95,500±27,439) and M4 (68,000±28,189) were significantly more than hCECs cultured in M1 (6,875±5,325 cells; ^*/†^ *p*<0.05) and M3 (11,594±5,117 cells; ^‡/§^ *p*<0.05) respectively (Table 3). The expansion of hCECs from P2 to P3 in M1 could not be carried out due to insufficient cell numbers. At the end of P3, hCECs cultured in M2 (57,125±14,250 cells) and M4 (36,875±12,691 cells) were found to be significantly more than cells cultured in M3 (1,750±1,848 cells; ^*/†^ *p*<0.05) respectively (Table 3). It should be noted that, although the absolute cell numbers obtained with the four different media differed greatly from donor to donor, a similar trend was observed across the four conditions.

**Table 3:** Expansion profile of hCECs in the four culture media.

Passage	Culture Medium	Total cell numbers±SD
0–1	[**M1**]	20,458±10,862^*/†^
	[**M2**]	123,625±11,813^*/‡^
	[**M3**]	26,208±16,335^‡/§^
	[**M4**]	102,833±35,534^†/§^
1–2	[**M1**]	6,875±5,325^*/†^
	[**M2**]	95,500±27,439^*/‡^
	[**M3**]	11,594±5,117^‡/§^
	[**M4**]	68,000±28,189^†/§^
2–3	[**M1**]	n.a.
	[**M2**]	57,125±14,250^*^
	[**M3**]	1,750±1,848^*/†^
	[**M4**]	36,875±12,691^†^

For passage 0–1 (*n* = 6), significance for non-parametric Kruskal-Wallis test with pair wise comparisons corrected for multiple comparison using Mann-Whitney U test was achieved between M1 & M2 (^*^ *p*<0.01); M1 & M4 (^†^ *p*<0.01); M2 & M3 (^‡^ *p*<0.01); M3 & M4 (^§^ *p*<0.01); For passage 1–2 (*n* = 4), significance was achieved between M1 & M2 (^*^ *p*<0.05); M1 & M4 (^†^ *p*<0.05); M2 & M3 (^‡^ *p*<0.05); M3 & M4 (^§^ *p*<0.05); For passage 2–3 (*n* = 4), significance was achieved between M2 & M3 (^*^ *p*<0.05); and M3 & M4 (^†^ *p*<0.05).

### Morphology of hCECs beyond P3

**Figure 5:**
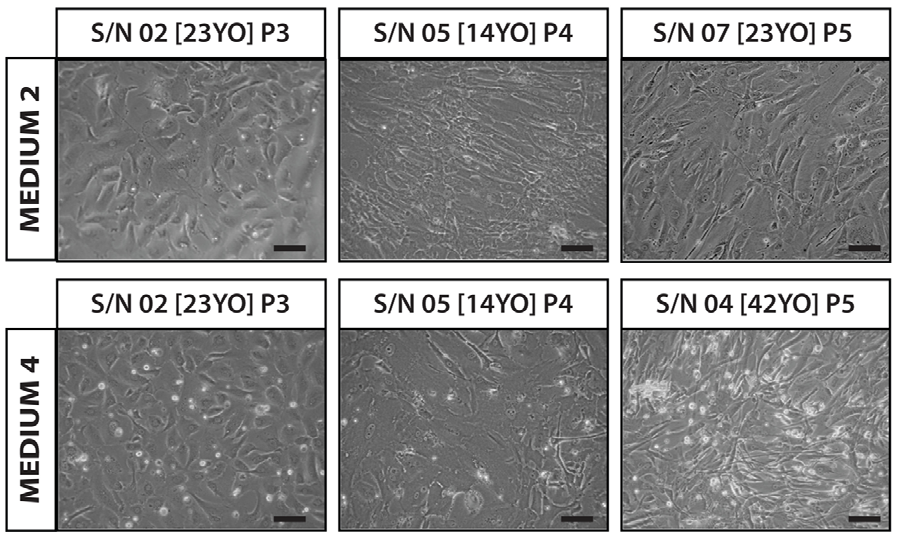
Morphology of cultured hCECs P3 to P5. Representative sets of photomicrographs showing morphology of hCECs at passage 3, passage 4 and passage 5 cultured in M2 and M4. (*n* = 6; Scale bars = 100 µm).

The expansion of hCECs in either M2 or M4 can be taken beyond P3. However, the cellular morphology of the hCECs changed within these cultures, some earlier than others (Figure 5). It appeared that the hCECs that were passaged beyond P3 in either M2 or M4 lost the unique polygonal morphology to take up an elongated morphology. In some cultures, the hCECs turned fibroblastic-like and appeared to have lost contact inhibition.

### Cultured P2 hCECs retained expression of ion channels Na^+^K^+^/ATPase and tight junction ZO-1

**Figure 6:**
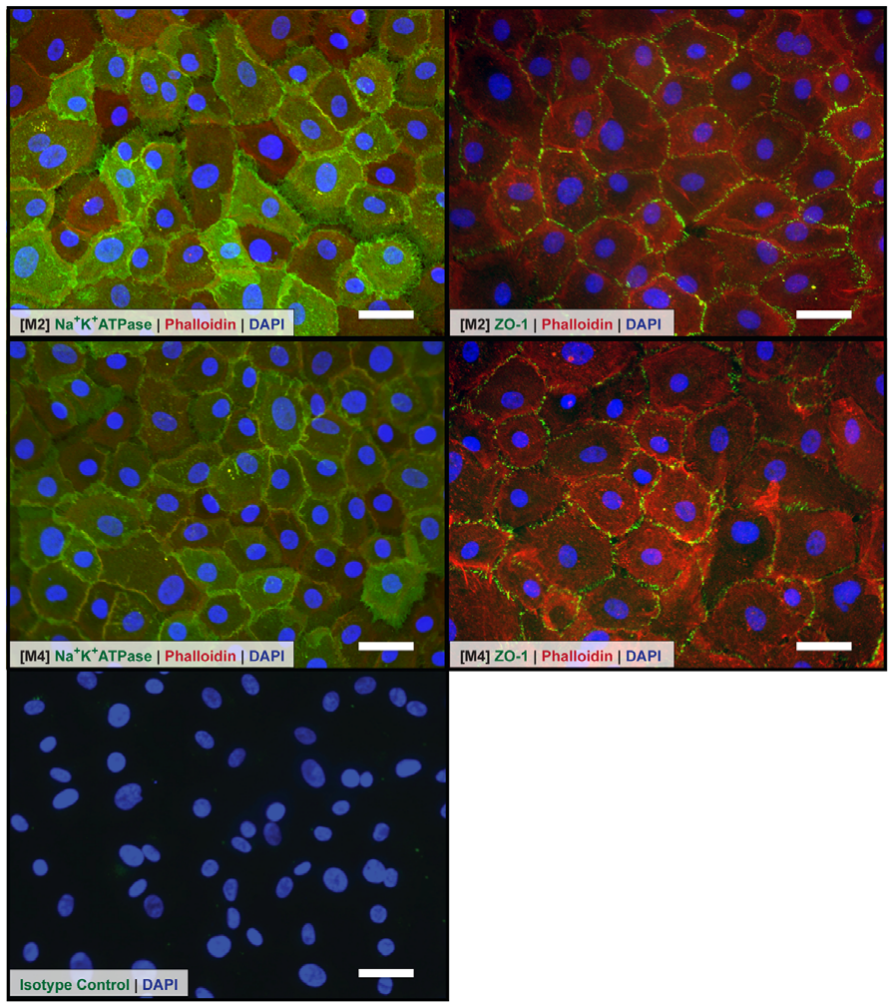
Expression of cultured P3 hCECs. Representative sets of photomicrographs showing expression of Na^+^K^+^/ATPase and ZO-1 by immunocytochemistry: Immunostaining of Na^+^K^+^/ATPase in **A**: M2 and **B**: M4. Immunostaining of ZO-1 in **C**: M2 and **D**: M4. Control staining **E**: Isotype matched IgG_1_ negative control. (*n* = 6; Scale bars = 50 µm).

The activity of Na^+^K^+^/ATPase is associated to the fluid pump function critical for the proper physiological control of corneal thickness by the corneal endothelium [16], [17]; whilst tight junction-associated protein ZO-1 is involved in the formation of focal tight-junction complexes important for the passive permeability properties of the corneal endothelial barrier function [4], [18]. Primary hCECs propagated in both M2 and M4 expressed Na^+^K^+^/ATPase and ZO-1 (Figure 6). Expression of Na^+^K^+^/ATPase displayed a ubiquitous staining pattern throughout the cell surfaces, and staining with ZO-1 showed distinctive staining towards the cell borders.

## Discussion

Proliferation of hCECs *in vitro* requires a complex mixture of supplements and growth factors. To date, a wide variety of serum-supplemented culture media, together with the use of various factors have been reported in the growth and proliferation of hCECs [2]. However, no direct comparison has been made between some of these culture media using hCECs isolated from a single donor to compare their capacity in supporting the isolation and long term cultivation of the hCECs. This is due to the low numbers of corneal endothelial cells obtainable per isolation. However, results obtained from such a side-by-side comparison not only negate significant donor variability, it enable a clearer evaluation of each culture medium, solely on their capacity to support the isolation, growth and subsequent expansion of hCECs established from individual donors.

Our approach uses isolated hCECs from pairs of research-grade donor corneas. The use of collagenase digestion to release the CE from the DM followed by another brief dissociation step using TE was to obtain smaller CE clusters and this generally enabled even seeding of the isolated hCECs into the four culture conditions. The application of TE also served to release the mitotic block within hCECs that are mediated by tight cell-to-cell contacts [19]. Although hCECs can be established directly onto uncoated cell culture plastic ware, the use of extracellular matrix such as FNC mixture significantly increased the attachment of isolated hCECs (Figure 2), consistent with a study reported by Engler and colleagues [20]. Overall, such a setup enabled the comparison of the four culture conditions (Table 1), and up to six culture conditions has been successfully compared using this approach.

The freshly plated hCECs adhered in each of the four culture conditions within four to six hours after seeding. It appeared that the corneal endothelial cells required approximately one to two days adapting to the different culture conditions. In this period of time the hCECs appeared morphologically similar in each of the four different culture media (Figure 3A to Figure 3D). The different growth dynamics of the hCECs became evident as the adherent hCECs proliferated in each of the culture medium over the next two weeks (Figure 3E to 3H). This is not surprising as the formulation of each medium was vastly different (Table 1). It became strikingly clear over the course of the study that hCECs cultured in either M1 or M3 were significantly less proliferative compared to isolated hCECs that were grown in either M2 or M4. Hence, hCECs grown in M2 and M4 reached confluence faster than in M1 or M3, usually within two and up to four weeks in some cultures. The variability we observed for cultured hCECs to reach confluence in the two proliferative media (M2 and M4) was partly due to possible variability in the overall yield during the isolation process, as well as donor sample variability. For example, hCECs derived from donors from a fatal motor vehicle accident were often found to grow better than cells derived from donor who had long-term chronic illness [21].

In our study, it appeared that M1 was unable to support the propagation of hCECs. The morphology of cultured hCECs that grew in M1 was mostly large and irregularly shaped. The proliferation rate and the amount of hCECs obtained at the end of P0 for M1-cultured hCECs were the lowest, and could not be expanded beyond the second passage. Comparatively, M1 was made from a more basic basal DMEM supplemented with the highest concentration of FBS (10%) but was not as heavily supplemented as the other three media assessed in this study with only bFGF (2 ng/mL) added. For hCECs that were expanded in M3, a more complex mixture of basal Ham's F12 and DMEM mixed at a 1∶1 ratio supplemented with 5% FBS amongst many other additives including EGF (see Table 1), majority of cells cultivated in this condition exhibited a homogeneous cellular morphology, but with large cytoplasmic volume. Proliferation rate of M3-cultured hCECs was slightly higher than that of M1-cultured cells, but was significantly lower when compared to either hCECs that were grown in M2 or M4. Total cell numbers obtained between P0 to P1 and P1 to P2 decreased dramatically, and in some cultures, could not be expanded beyond the second passage. Although extended propagation of hCECs could not be achieved in either M1 or M3, it should be noted that our isolation protocol and culture methodologies varied from previous reports using these media [12], [14]. In the study where M1 based medium was reported, Ishino et al. [12] stripped the corneal endothelium from the peripheral corneoscleral tissue, dissociated the hCECs using dispase and cultured the isolated CECs on collagen IV-coated culture plate. With these, they were able to obtain confluent monolayer of cultured hCECs at the fifth passage following a 1∶2 to 1∶8 splitting ratio every 10–20 days [12]. The medium reported by Li et al, [14] M3, also isolated hCECs from the peripheral corneoscleral tissue, and demonstrated that hCEC aggregates isolated could be cultured as a spheroid culture in a high-calcium, serum-free medium for 3 weeks. Although aggregates preserved in the serum-free medium could yield a monolayer of hexagonal hCECs, multiple passages of hCECs grown in M3 were not reported in their study.

Both M2 and M4 were able to support the continual expansion of hCECs. Interestingly, hCECs isolated from different donors appeared to have different preferences to the two proliferative media: some isolated hCECs grew well in M2 whilst some grew better in M4. For example, two primary cultures derived from a 34-year-old donor (Figure 3N and Figure 3P) and a 22-year-old donor (Figure 3R and Figure 3T) grew better in M2 when compared to M4 in terms of their morphology. However, in the third set of hCECs derived from a 14-year-old donor (Figure 3J and Figure 3L), as early as P0, hCECs established in M2 proliferated rapidly and appeared to have lost its unique morphology and contact inhibition. On the contrary, M4-cultured hCECs from the same 14-year-old donor exhibited an almost homogeneous layer of tightly packed polygonal cells. As M2 and M4 were formulated from different basal media each supplemented with different growth factors and additives, we were not able to objectively speculate on the medium preferences exhibited by hCECs isolated from different donors.

The established hCECs that were expanded to the third passage in either M2 or M4 expressed characteristic markers indicative of the corneal endothelium, such as tight junction associated protein ZO-1, and sodium-potassium pump enzyme Na^+^K^+^/ATPase. Although hCECs cultivated in these two media can be taken beyond the third passage, in most cases, the classical morphological integrity of polygonal cell-shape could not be maintained in our study (Figure 5).

A limitation of our study was that the propagation of isolated primary hCECs in the four culture media were performed using the ‘peel-and-digest’ methods reported in this study, which differed from the isolation strategies used within the original studies of the four hCEC-culture systems cited. Hence, the comparative outcomes from the four culture media and observations reported in this study could not be drawn as a direct comparison to the original studies where each culture medium was reported. This is because other important factors of the cell culture processes, such as the cell digestion strategy, and the usage of specific culture substrates to aid the adherence of hCECs were used. These factors may have significant influence on the proliferation and morphology of isolated hCECs. However, the hCECs isolation methodology, using a series of paired donor corneas from single donors, presented in this study enabled a valid assessment to be made between various hCEC culture media whilst negating a key confounder: donor-to-donor variability. Together with the medium that produced the most favorable outcome, a robust and consistent platform for the isolation and *in vitro* propagation of hCECs can be established and further improved systematically.

### Conclusion

For the development of a suitable alternative donor graft material through tissue engineering, it is imperative that a robust system for the isolation and propagation of cultivated hCECs can be established. In this study, we described a relatively simple, yet systematic hCECs isolation protocol from both corneas of an individual donor. We showed that the isolated hCECs can be established in all four media for a short period of time, but only in this study, only two of the media, M2 and M4, were able to support the continual propagation of hCECs. Furthermore, a differential preference was observed where some isolated CECs grew better in M2, and some in M4. However, most cultivated hCECs lose the unique structural morphology of corneal endothelium after a few rounds of passages, some sooner than others. It is unclear as to what are the factors that contributed to the differential preference of culture medium or the observed cellular changes after several rounds of passages. However, it is not surprising that the complexity of each culture medium, together with donor-to-donor variability play a role in the changes observed. Hence, future work that utilizes cultivated hCECs for the development of a cell-tissue engineered corneal graft alternative should be performed at the third passage or before the cultivated hCECs lose their characteristic cell morphology.

## Materials and Methods

Dulbecco's modified Eagle's medium (DMEM), OptiMEM-I, DMEM/Ham's F12, Ham's F12, Medium 199 (M199), fetal bovine serum (FBS), bovine pituitary extract (BPE), Dulbecco's Phosphate-Buffered Saline (PBS), TrypLE™ Express (TE), gentamicin, amphotericin B, penicillin & streptomycin were purchased from Invitrogen (Carlsbad, CA, USA). Dimethyl sulfoxide (DMSO), ITS (Insulin, transferrin, selenium), hydrocortisone, ascorbic acid, calcium chloride, chondroitin sulphate, cholera toxin, Trypan Blue solution (0.4%) were purchased from Sigma (St. Louis, MO, USA). FNC Coating Mix^®^ was purchased from United States Biologicals (Swampscott, MA, USA). Collagenase A was purchased from Roche (Mannhein, Germany).

### Ethics Statement

The following protocols conformed to the tenets of the Declaration of Helsinki, and written consent was acquired from the next of kin of all deceased donors regarding eye donation for research. The study was approved by the institutional review board of the Singapore Eye Research Institute/Singapore National Eye Centre.

### Research-grade Human Corneoscleral Tissues

A total of 20 pairs of research-grade corneoscleral tissues from cadaver human donors considered unsuitable for transplantation with endothelial cell count of >2,000 were procured from Lions Eye Institute for Transplant and Research Inc. (Tampa, FL, USA). Overall general health of the donor before death was also considered which included previous history or medical treatment that might damage or affect the growth of the corneal endothelium [13]. Research corneas were preserved and transported in Optisol-GS at 4°C, and were used within 13 days from preservation. The ages of donors ranged from 10 to 42 years (Table 4).

**Table 4:** Donor information.

Serial Number	Age	Sex	Days to Culture	Cell Count (OS/OD)	COD	Experiments				
						A	B	C	D	E
01	34	M	5	3086/2825	MVA	•	•			
02	23	M	11	3058/3077	Blunt Trauma	•	•			
03	33	M	7	2865/2976	Acute Cardiac Crisis	•	•			
04	42	M	8	2639/2660	Acute Cardiac Crisis	•		•		
05	14	M	12	2907/3215	Acute Cardiac Crisis	•				
06	37	M	10	2646/2674	Acute Cardiac Crisis	•				
07	23	F	8	3012/3049	Overdose	•	•		•	
08	14	M	7	3344/3636	Acute Cardiac Crisis	•	•		•	
09	22	M	5	2841/2632	Cystic Fibrosis	•	•		•	•
10	38	M	12	2494/2481	Multiple GSW	•				
11	10	F	12	3745/3448	Cerebral Palsy	•				•
12	24	F	12	2564/2506	Acute Cardiac Crisis	•				•
13	34	M	6	2874/2770	Acute Cardiac Crisis	•			•	
14	19	M	8	3378/3257	MVA	•			•	•
15	28	F	3	3559/3509	Cardiopulmonary Arrest	•				•
16	18	M	7	3279/3106	MVA	•			•	•
17	19	M	6	2506/2463	Trauma Gunshot	•			•	
18	28	M	9	2597/2732	Overdose	•			•	
19	34	F	12	3106/2882	Overdose	•	•	•		
20	30	M	13	2882/3145	Acute Cardiac Crisis	•	•	•		

COD: cause of death. Donor age ranged from 10 year-old to 42 year old with a median age of 26 year old. Days taken from death of donor to the initiation of corneal endothelial cell culture ranged from 3 days to 13 days with a median of 8 days. Experiment A: morphological assessment/growth profile - P0 to P1; Experiment B: morphological assessment/growth profile - P1 onwards; Experiment C: Cell adherence analysis – xCelligence; Experiment D: Cell proliferation – Click-iT EdU; Experiment E: Immunofluorescence staining.

### Isolation and Growth of Human Corneal Endothelial Cells

Research corneas were incubated in three washes of antibiotic/antimycotic solution in PBS, 15-minute each. Primary cultures of hCECs were established as described. Primary hCECs were isolated using a two-step, peel-and-digest method. Corneoscleral rims were placed endothelial-side-up on a disposable cornea vacuum punch (Ripon, England), and mildly stabilized by the vacuum suction created (Figure 2A). A brief 30 seconds treatment with Trypan Blue solution (0.2%) was used to delineate Schwalbe's line. The DM-endothelial layer was carefully stripped off, approximately 1 mm anterior to the Schwalbe's line (away from the trabecular meshwork) from the posterior stroma under the dissecting microscope (Nikon, Japan). Paired DM-endothelial layers obtained were pooled and digested enzymatically in collagenase A (2 mg/ml) for at least 2 hours and up to 6 hours. This allowed full detachment of the CE from the DM, which tended to conglomerate into tightly-packed hCEC clusters. The hCEC cultures were rinsed once in PBS and further dissociated in TE for 5 minutes. Cell pellets collected after a mild centrifugation (800 *g* for 5 minutes) were plated equally into organ-culture dishes coated with FNC coating mixture, in four culture conditions coded as M1, M2, M3, and M4 (Table 1). All incubation and cultivation of hCECs were carried out in a humidified incubator at 37°C containing 5% CO_2_. Fresh media were replenished every two days.

After primary cultures of hCECs reached confluence at P0, cells were dissociated using TE, and sub-cultured on FNC-coated culture dishes at a matched plating density of 5,000 cells/cm^2^. Subsequent passages of hCECs (P1 through to P3) were also dissociated using TE. During the course of the study, cultures with insufficient cell numbers for subsequent passage were excluded. A Nikon TS1000 microscope with a Nikon DS-Fi1 digital camera was used to capture phase contrast images during expansion and at confluence to document general hCEC morphology. Variation in hCEC size (polymegathism) and the variation in cell shape (pleomorphism) of confluent cultures at P0 were assessed using Nikon NIS-Elements basic research software (Nikon, Japan). In each culture condition, the mean and standard deviation of cultivated corneal endothelial cell sizes were calculated, from which a coefficient of variation index in cell area (SD/mean cell area X 100) was calculated. The closer the calculated index value was to zero, the more uniform the overall cell sizes were and vice versa [22].

### Real-Time Cell Adherence Analysis

The adherence of cultured hCECs, with and without FNC coating, was assessed using the xCelligence real time cell analyzer (Roche Diagnostics GmbH, Penzberg, Germany). The study was performed according to manufacturer's instructions. Cultured hCECs at P1 were seeded at 2,000 cells per well in an E-Plate 96 (Roche) in quadruplicates. The seeded cells were equilibrated for at least 30 minutes in the tissue culture incubator before electrode resistance was recorded. Cell adherence and their subsequent growth were monitored for up to 24 hours.

### Cell Proliferation Assay

Proliferation of the corneal endothelial cells were assessed using a 5-ethynyl-2′-deoxyuridie (EdU) incorporation assay, by using a Click-iT EdU Alexa Fluor 488 cell proliferation assay kit (Invitrogen, Carlsbad, CA, USA). Cultured CECs were passaged using TE and seeded in their respective culture conditions (Table 1) on FNC-coated glass slides at a plating density of 5,000 cells/cm^2^ for 24 hours. The cells were then incubated in their respective medium containing EdU (10 µM) for another 24 hours. Corneal endothelial cells were washed with PBS, and fixed using 4% paraformaldehyde (PFA) for 15 minutes on ice, followed by a 0.1% Triton X-100 in 3% BSA block and permeabilization step for 20 minutes, all at room temperature. Incorporated EdU was detected by fluorescent-azide coupling Click-iT reaction [23]. Briefly, cells were incubated for 30 minutes in a reaction containing azide-conjugated Alexa Fluor 488 dye in 1× Click-iT EdU reaction buffer supplemented with 4 mM CuSO_4_. Cells were washed twice with 1 mL 3% BSA in PBS, and mounted on glass slides with Vectorshield containing DAPI (Vector Laboratories, Burlingame, CA, USA). Images of cells were examined using a Zeiss Axioplan 2 fluorescence microscope (Carl Zeiss, Germany). At least 500 nuclei were analyzed per experiment and data point.

### Antibodies and Immunofluorescence

Cells cultured on glass slides or glass coverslips were fixed in either 100% ice-cold ethanol for 5 minutes, or freshly prepared 4% PFA on ice for 20 minutes. Ethanol-fixed cells were immersed in a PBS block solution containing 10% normal goat serum. PFA-fixed cells were permeabilized in 10% block solution containing 0.1% Triton X-100, both for 30 minutes at room temperature. Following this, the samples were incubated with primary and subsequently, secondary antibody (in the dark), each for 1 hour at room temperature. Between incubation, cells were washed twice with PBS. Labeled cells were mounted onto coverslips in Vectorshield containing DAPI (Vector Laboratories). The following primary antibodies were used: mouse IgG_1_ anti- Na^+^K^+^/ATPase α1 (5 µg/mL; Santa Cruz Biotechnology), mouse IgG_1_ anti-ZO-1 (5 µg/mL; BD Biosciences Pharmingen), and rhodamine conjugated anti-phalloidin (0.5 µM; Invitrogen). The secondary antibody used was Alexa Fluor 488 goat anti-mouse IgG (2 µg/mL; Invitrogen). Negative controls were cells incubated with an anti-mouse IgG_1_ isotype control (5 µg/mL; BioLegends) in place of the primary antibody.

### Statistics

All numeric data obtained were expressed as mean ± standard deviation. Differences in the values of cell indexes (Figure 2I) were analyzed using independent sample *t*-tests. Comparisons of hCECs sizes cultured in the four media (Table 2) were performed using two-way ANOVA followed by post-hoc Bonferroni test for multiple comparisons. Absolute cell numbers obtained from the different conditions during each of the 3 passages (Table 3) were analyzed using a non-parametric Kruskal-Wallis test with pair wise comparisons corrected for multiple comparisons done using Mann-Whitney U tests. Finally, the analysis of the cell proliferation using Click-iT EdU assay was evaluated by the means of the chi-squared test with Yates' collection. Values were deemed to be significant when a significance level with a *p*-value of less than 0.05 was achieved.
